# Using Cooking Schools to Improve the Pleasure of Food and Cooking in Patients Experiencing Smell Loss

**DOI:** 10.3390/foods13121821

**Published:** 2024-06-10

**Authors:** Alexander Wieck Fjaeldstad

**Affiliations:** 1 Flavour Clinic, University Clinic for Flavour, Balance and Sleep, Department of Otorhinolaryngology, Regional Hospital Gødstrup, Hospitalsparken 15, 7400 Herning, Denmark; alefja@rm.dk; 2 Flavour Institute, Department of Clinical Medicine, Aarhus University, Palle Juul-Jensens Boulevard 99, 8200 Aarhus N, Denmark; 3 Center for Eudaimonia and Human Flourishing, Linacre College, University of Oxford, Stoke House, Oxford OX3 9BX, UK

**Keywords:** olfaction, rehabilitation, hyposmia, anosmia

## Abstract

Smell loss affects around 15–20% of the population, with a major effect on the quality of life. The most common complaint is the impairment of the eating experience, with around 90% of patients reporting this issue. A study conducted at a specialised Taste and Smell Clinic investigated if food and cooking can positively affect the enjoyment of food, subjective cooking skills, and quality of life in patients with smell loss. The 49 participants in the study received a 5-week cooking school course that focused on emphasizing the other senses to regain the enjoyment of food. Participants gained more confidence in cooking, and their quality of life improved significantly. Positively evaluated recipes were adjusted based on feedback and published as free e-books in Danish, German, and English. Eating and cooking are multisensory experiences, and the perception of food depends on the complex interaction of senses and surroundings. If the olfactory input is reduced or absent, both the enjoyment and cooking experience can be negatively affected. Therefore, focusing on food and cooking can have a positive impact on patients with smell loss.

## 1. Introduction

While many people have their morning coffee or tea without giving it much thought, these daily routines have changed drastically for many in the wake of COVID-19. Olfactory loss was not only a dominant symptom during the acute phase, but is often a long-lasting or permanent burden [1]. Even before COVID-19, losing the sense of smell was a common hidden handicap, with approximately 15% of the population suffering from a reduced sense of smell and 2–3% suffering from a complete smell loss [2,3].

This sudden change in awareness of the existence of smell loss among the general public was accompanied by an increase in research efforts to treat smell loss worldwide. Despite these efforts, olfactory training with essential oils remains the cornerstone for treating smell loss in most patients [4]. While some patients regain olfactory function through olfactory training and/or spontaneous recovery, a large proportion of patients experience either partial or no recovery, which often negatively impacts their quality of life (QoL) [5,6].

Of all the negative aspects affecting patients with smell loss, such as various social challenges, risk of food poisoning, and other hazards, the most common complaint is impairment of the eating experience, affecting 90% of patients [7], while 73% complain of problems with cooking [8]. These large negative effects on the enjoyment of food and cooking are related to the dominant role of olfaction in the overall flavour experience [9].

Eating and cooking are truly multisensory endeavours. Apart from orthonasal and retronasal aromas sensed by the olfactory system, the gustatory system contributes with information on the five basic tastants, and the trigeminal system provides information on the mouthfeel, including temperature, somatosensation, and pain. Hearing provides not only information on texture, such as crispiness during mastication, but also more discrete information from, for example, carbonated beverages. Additionally, vision is important for evaluating edibility and setting up expectations for the other senses. In addition to these food-related sensory inputs, numerous other internal and external factors influence our perception of food, such as hunger, mood, atmosphere, and social context [10]. The perception of food depends on the complex interaction of both the senses and surroundings, where the overall perception can go far beyond the sum of its parts [11]. If one single factor changes, it can have enormous effects on the hedonic yield, e.g., a decarbonated soda, a warm wine, a cold steak, or a brown-spotted apple [10]. If the olfactory input is reduced or absent, both the overall perception of food and the remaining sensory inputs are often severely altered [12].

This results in several challenges during cooking, where enjoyment of cooking, confidence in one’s abilities, and ability to accomplish desired results are significantly reduced in patients with smell loss [13].

No prior studies have systematically investigated methods to improve cooking and eating in patients with smell loss in settings where patient feedback and hands-on training have been key factors. In the setting of treating and consulting patients, this results in a lack of tested and tangible advice on improving cooking abilities and enjoyment of food following smell loss.

This study aimed to evaluate the effects of a novel approach to improve the food-related quality of life and cooking abilities in patients experiencing smell loss. By describing the modification of recipes and pedagogical considerations in creating the cooking schools, a further aim is to enable other researchers and clinicians to create similar courses for patients with smell loss.

## 2. Materials and Methods

### 2.1. Participants

In total, 49 adult participants with olfactory dysfunction and smell-related issues who took pleasure in eating or cooking were included, primarily from our Taste and Smell Clinic at Gødstrup Regional Hospital, Denmark [14]. Participants with all aetiologies of olfactory dysfunction were included. Participants were offered a free 5-week cooking school course focusing on emphasizing the other senses to regain the enjoyment of food. Inclusion criteria included having an intact sense of taste, which was confirmed by taste testing before start-up by either correct recognition of sweet, salty, sour, and bitter taste sprays [15] or, in case of an incorrect taste identification of taste sprays, completion of the taste drop test within the normal range for all four tastants [16]. As umami generally has a low identification rate in a normative population, this basic tastant was excluded from this testing [17]. For participants without recent olfactory and gustatory testing (within two months) from our Taste and Smell Clinic, olfactory and gustatory testing were performed before starting the cooking school.

As the cooking school required a high degree of acceptance of ingredients and extended presence in a room with cooking odours, only patients with a mild severity of subjective parosmia were allowed to participate in the cooking schools.

The study was performed following the Declaration of Helsinki’s ethical principles for medical research. Approval to conduct the study was granted by the Regional Ethical Committee, Denmark Central Region (1-10-72-274-21).

### 2.2. Testing of Olfactory Function and Questionnaires

Olfactory testing (Sniffin’ Sticks TDI-score, Burghart Messtechnik GmbH, Pinneberg, Germany) was carried out in all participants before start-up. This olfactory test has been validated for a Danish population [18,19,20].

Participants filled in questionnaires before the first session, the day after the last session, and three months after the last session. These questionnaires included subjective sensory function, food-related quality of life (QoL), and food and cooking habits. Cooking habits were evaluated by applying the Cooking and Food Provisioning Action Scale (CAFPAS) [21]. Food-related QoL (‘Please rate how food impacts your quality of life’; 0 = ‘most pronounced negative impact possible’, 100 = ‘most pronounced positive impact possible’) was evaluated using a visual analogue scale (VAS) (0–100).

### 2.3. Preparation and Modification of Recipes

Initial pilot testing of concepts was initiated in 2016, where all staff at a collaborating restaurant wore nose clamps for 14 consecutive days to gain an understanding of the effects of smell loss on sensory perception of the food. This led to the first creation of meals targeted at patients with smell loss, which later aired on Danish national television in 2018 [22]. In collaboration with a neurogastronomy chef [23], this initial pilot program relating to cooking and knowledge on food perception and cooking habits in patients with olfactory dysfunction [13] was used to create initial cooking school recipes. These recipes were modified and supplemented by additional recipes during the following cooking schools through participant feedback. All participants filled in a questionnaire on each recipe, rating difficulty, usefulness of the taste and texture kit, willingness to make the dish at home, and usefulness of the recipe for enhancing other recipes. All dishes were further evaluated during the social dining after cooking, where the taste kit was available to improve balance and consensus among participants on the relevance and edibility of the recipe was reached. For recipes with overall low ratings (<50) or occurrence of parosmic triggers among several participants, the recipe was discarded. For dishes with overall moderate ratings (50–75), recipes were modified by the chefs based on participant feedback before usage in later courses. For dishes with overall high ratings (>75), recipes were reused in a later course, where subsequent overall high ratings (>75) would result in the inclusion of the recipe in the cookbook. For further details on selected recipes, see the results section.

### 2.4. Cooking School Contents and Structure

The five-week course consisted of weekly sessions with theory, practical cooking guided by chefs, social dining, and evaluation of recipes, see Table A1 (Appendix A).

Through combined theory and practice during the cooking schools, the ability to season the food with basic tastants and highlight the flavour enhancement of the food with texture, temperature, tactility, and trigeminal stimulation was trained.

Seasoning with basic tastants was trained using a ‘Taste Kit’, consisting of ingredients with different basic taste properties (e.g., honey (sweet), tamari/soy sauce (umami), vinegar (sour), salt (salty), and fresh herbs/lettuce (bitter)). A taste kit and the newest version (continuously updated) of our cookbook [24] were distributed to all participants on the first day of the cooking school. Participants were encouraged to use these flavour enhancement methods at home between sessions. As most ingredients in the taste kit can be stored at room temperature, participants were recommended to place the taste kit in their kitchen next to the stove and use it daily.

The cooking school was structured to give the participants the necessary knowledge, training, and feedback to empower their abilities to cook and the confidence to apply the methods of their training beyond the recipes used in the cooking school, see Table A2 (Appendix A).

### 2.5. Statistics

Data were analysed using JMP Pro 16 (SAS Institute, Cary, NC, USA). Normal distribution was assessed using QQ plots and the Shapiro–Wilk W test. For parametric data, mean values were compared using the Student’s *t*-test and presented as means with a 95% confidence interval (CI). For analysis of individual differences over time, the matched pair *t*-test was applied.

Fisher’s Exact Test was used to compare ordinal variables between groups, where contingency tables included fields of observations below five. One-way ANOVA and Spearman’s rho were used to analyse correlations. The α level of statistical significance was set at 0.05.

## 3. Results

### 3.1. Demographics

**Figure 1:**
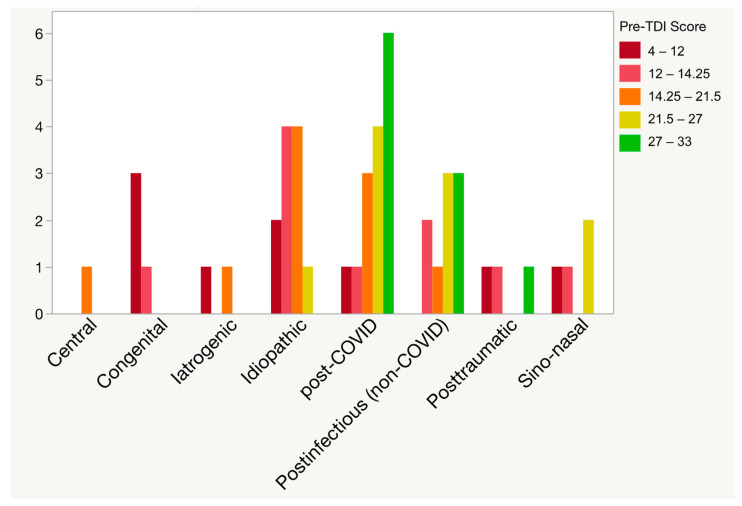
Aetiology and severity of smell loss. Participants had subjective smell loss and smell-related challenges with the enjoyment of food and/or cooking. TDI: composite Sniffin’ Sticks olfactory test score (threshold, discrimination, and identification).

The cooking school participants of the five classes were predominantly female (75%), had a wide age span, had a long duration of smell loss, and differed in aetiology and severity of smell loss (see Table 1 and Figure 1).

### 3.2. Recipes

After pilot testing, the recipes had a focus on texture, temperature differences, strong aromas, and some with a relatively high content of umami and trigeminal spiciness. These three last components were represented, for example, in Asian-inspired dishes with miso soup (umami) and dishes with high contents of fresh herbs (highly aromatic). During the first two cooking school classes, oral and written evaluations of the dishes with strong aromatic contents of fresh herbs were continuously low due to the bitter taste of fresh herbs. Ratings were similarly low for dishes with high umami and trigeminal contents in comparison with similar dishes with a balanced composition of the five basic tastants and less pronounced (yet present) spiciness, see Table 2.

**Figure 2:**
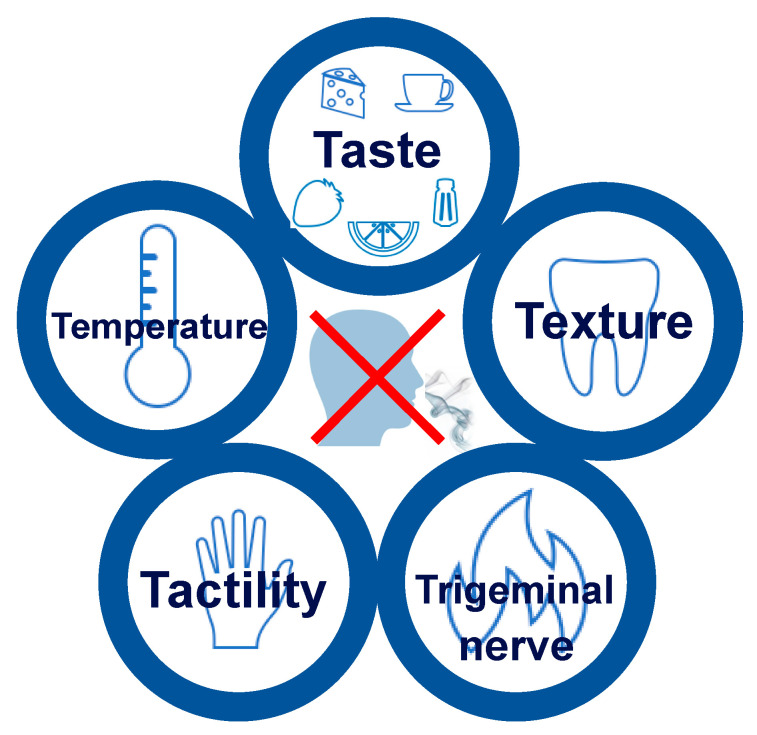
Sensory elements for flavour enhancement in patients with smell loss. “The 5T’s” are taste, texture, temperature, tactility, and trigeminal nerve. Reprinted with permission from AW. Fjaeldstad, “Cooking With a Smell Loss”; published by Apple Books, 2023” [24].

Recipes were tested and modified to include “Four T’s”, consisting of texture, temperature, trigeminal pain, and tactility. As the importance of balancing basic tastants became clear in both the oral and written evaluations, this method was renamed “The 5T’s” to include “taste” after the third cooking school class, see Figure 2.

Positively evaluated recipes were adjusted according to participant feedback, re-evaluated, and if high ratings persisted, recipes were published in an online cookbook in Danish, English, and German [24].

### 3.3. Cooking Problems and Habits

The CAFPAS questionnaire on cooking and provisioning revealed several significant changes in habits and attitudes following the cooking school. In general, there were improvements in almost all aspects of cooking, where scores of participants with smell loss after the cooking school changed towards reference scores of a normosmic population. For the most pronounced improvements, see Table 3 (all scores are included in Table A3). The ratings after the cooking school of “Confidence in choosing between similar products” and “Involvement in daily meal preparations” were even higher than the normosmic controls.

### 3.4. Satisfaction with the Cooking School and Parosmia

Overall ratings of satisfaction with the cooking school were comparable between participants without parosmia and participants with mild parosmia (*n* = 16) (scale 1–5; mean scores 4.78 vs. 4.76; *p* = 0.7111).

Overall ratings of satisfaction with the cooking school were significantly higher after modification of the methods (focus on “the 5T’s”) and recipes (after the second cooking school class) (scale 1–5; mean scores 4.53 vs. 4.93; *p* = 0.0122).

### 3.5. Food-Related QoL

Food-related QoL increased following the cooking school and remained significantly higher at follow-up three months after the cooking school (see Table 1). While there was a significant difference from before the cooking school to the end (*p* = 0.0120) and three months after the cooking school (*p* = 0.0269), there was no significant difference in rating from the end of the cooking school to three months after the cooking school (*p* = 0.6844).

## 4. Discussion

### 4.1. Impact on Cooking Habits and Food-Related QoL

As a consequence of smell loss and the associated challenges of cooking, patients decrease their engagement and confidence in cooking [13]. While the negative effects on appetite and behaviour have been addressed [5,7,13,25], neither systematically tested advice nor interventions concerning cooking have previously been published. As such, the current findings of significantly improved cooking habits and improved ratings of food-related QoL indicate a potential for improvement of clinical care for patients with smell loss who complain of reduced enjoyment of food or cooking. As this constitutes the vast majority of patients with smell loss [5,7], there is a need for more multidisciplinary approaches to olfactory rehabilitation with a focus on food. Furthermore, the improved ratings after the cooking school on several aspects of the CAFPAS questionnaire even exceeded the normosmic ratings. This gives interesting perspectives on a potentially more general possible benefit of attending the cooking school on cooking habits.

### 4.2. Flavour Enhancement of Food in Patients with Olfactory Deficits

As a result of the high occurrence of olfactory and gustatory impairments during and after the COVID-19 pandemic [26,27,28,29], new insights into the dynamics of altered eating behaviour have emerged [30]. Profound effects of olfactory loss on appetite, sensory perception, and food behaviour have been described [25]. Similar to our findings, increased focus on texture and trigeminal stimulation may be beneficial for maintaining food-related pleasure [31]. However, after their smell loss, most patients do not fully utilize other flavour-related senses in cooking or eating [13]. This may contribute to the heterogeneous reports on the influence of olfactory loss on dietary behaviours [32].

Our novel rehabilitation method for patients with smell loss focused on using the “5T’s” to ensure adequate attention to taste, texture, temperature, tactility, and trigeminal nerve stimulation in the food. The usage of this method was the cornerstone in every session and evaluation of the food, presentations of prepared meals, and the ‘food-jam-assignment’ during the last session where no recipe was available. While the theoretical advantages of flavour enhancement have previously been mentioned in the literature [33], no tested or complete guide to cooking Is currently available for patients or clinicians. Based on the “5T’s” the cooking school improved cooking habits and abilities in participants with smell loss towards ratings comparable with normosmic controls. Following the five-week course, participants increased involvement in daily meal preparations and to a higher degree preferred to cook rather than have food prepared. Cooking was rated as a more fulfilling activity after the cooking school. As food and cooking-related problems are consistently rated among the highest complaints in patients with smell loss [7,34], these findings indicate that targeted efforts on flavour enhancement can alleviate some of the negative consequences of smell loss.

### 4.3. Parosmia

A further hindrance to the enjoyment of food is the occurrence of distorted olfactory perception (parosmia). Recently, new insights have emerged indicating specific molecular patterns of odorant triggers of parosmia [35]. These not only include groups of foods, but parosmic triggers are also released during common preparations of a meal, such as frying and baking [36]. While the setup of the current study with extensive cooking in a confined space during the cooking school constrained the inclusion of participants with severe parosmia, we found comparable high evaluations of satisfaction between participants with and without mild parosmia. Close adherence to the avoidance of common parosmic triggers described by Parker et al. [35,36], indicates promising prospects for applying similar methods of flavour enhancement in future cooking schools designed specifically for parosmic participants.

### 4.4. Gustatory Impairment in Patients with Olfactory Dysfunction

A solid understanding of the senses contributing to flavour is essential for an adequate understanding of patients’ sensory abilities and guidance of patients.

In the clinical setting, patients with olfactory loss often complain of an affected sense of taste despite taste scores within the normative range [37]. While this well-known phenomenon of taste–smell confusion may be related to a linguistic conundrum where taste is generally used to describe the combined sensation of flavour [38], it may also be affected by a decrease in gustatory function following an acquired olfactory impairment [39].

In the current study, the correct identification of supra-threshold tastants was a prerequisite for participation. However, a recent study on taste rehabilitation has indicated that gustatory sensitivity can be increased through taste training [40]. As such, some patients may benefit from focused gustatory training before engaging in cooking using “The 5T’s”. On the other hand, the continuous focus on taste seasoning with basic tastants may have similar positive effects on gustatory sensitivity as taste recall training. However, this was beyond the scope of this current study.

### 4.5. Future Perspectives

The current study investigated the effects on unselected participants with olfactory loss and affected enjoyment of food and cooking. However, specific patient groups with high occurrence of olfactory loss and special dietary requirements may have even more pronounced benefits from similar interventions. These groups include, for example, patients with chemosensory deficits following radio-/chemotherapy, where appetite and weight loss are key indicators for successful outcomes [41,42,43,44], dietary habits, and diet adherence in patients with diabetes [45,46,47,48], patients with dietary requirements associated with kidney disease [49,50,51], and elderly patients in general, where enjoyment of food may become severely affected due to the decline of chemosensory function [52,53,54,55].

As food-related cultural backgrounds and preferences may differ in other countries or cultures, confirmatory studies are needed to investigate the effects of these methods in different populations.

Further studies are needed to optimise the use of cooking as smell loss rehabilitation. These studies should include validated olfactory testing to explore if the sensory stimulation during increased involvement in cooking could have similar effects as other forms of olfactory training.

### 4.6. Limitations

This study describes the initial findings from a smell loss rehabilitation initiative. Due to the aim of describing the establishment of the methods applied in this novel project, alterations of methods and recipes were made during the process, e.g., the change from “4T’s” to “5T’s”. As described in the methods section, this study included data from the first two classes of the cooking school, where the concept and methods were, to a high degree, still under development. As such, data on follow-up and the effects of the interventions in this manuscript may be affected by the changes made during the initial phases of this novel approach to relieving the negative consequences of olfactory dysfunction. Nonetheless, these preliminary findings have shown significant positive results for both QoL and cooking habits, despite the lower level of satisfaction ratings during the first two courses. The purpose of this manuscript was not only to investigate the feasibility and possible effects of the cooking schools, but also to describe the process of planning and conducting food-related rehabilitation initiatives for clinical and culinary individuals to ease the process of conducting similar initiatives. As the aim was to establish a framework for conducting cooking schools, we have not included a control group in this current study.

The assessment of food-related QoL relied solely on a single question posed to participants: “Please rate how food impacts your quality of life”. While this approach provides a broad perspective on the specific impact of food, it might oversimplify the multifaceted nature of QoL. Future studies on the impact of food on the QoL in patients with smell loss could benefit from applying validated questionnaires covering both food and other domains, such as the TASTE questionnaire [56].

## 5. Conclusions

A framework for a cooking school for patients with smell loss was established and evaluated. This study found that cooking schools for patients with smell loss significantly improved cooking habits, cooking skills, and food-related QoL. The participants’ cooking habits and skills increased to levels near the normosmic reference group and, for some aspects, even exceeding the normosmics after the five-week cooking school.

Recipes and cooking methods are freely available in online cookbooks in Danish, German, and English. Although problems related to food and cooking are among the most common complaints in patients with smell loss, these are the first comprehensive food-related methods and advice that have been systematically tested by patients with smell loss. This may benefit both patients and clinicians as an inspiration for future efforts to alleviate the negative consequences of olfactory loss.

More studies are needed to further investigate the potential of cooking as rehabilitation in patients with olfactory dysfunction.

## Figures and Tables

**Table 1:** Demographics and changes in QoL assessment.

	Before Cooking School	End of Cooking School	3 Months after Cooking School	Δ	*p*-Value
Age (Mean, IQR *, range)	57 (IQR 50–67; range 20–79)			-	-
Gender (female *n* (%))	37 (75%)			-	-
Duration of smell loss (Mean, IQR *, range)	88 months (IQR 22–69; range 9–700)			-	-
Food-related QoL (*n* = 48) (range 0–100)	58.2	68.7	66.6	10.5 8.4	0.0120 0.0269

* IQR: interquartile range has been added to give further details on the distribution.

**Table 2:** Examples of recipe evaluations (mean scores), group consensus, and actions.

Recipe	Difficulty (High (0)–Low (100))	Taste Kit Usage (Low (0)–High (100))	Make at Home (Low (0)–High (100))	Overall Learning (Low (0)–High (100))	Group Consensus	Recipe Action
**Fried duck with Asian sauce (*n* = 9)**	41	32	52	62	Too spicy and overwhelming umami	Discard
**Beetroot carpaccio** **(*n* = 10)**	81	78	83	91	Off flavour for some with raw beetroot, slight parosmic trigger. Texture not appreciated	Change beetroot to mushroom and re-evaluate
**Baked pumpkin** **(*n* = 9)**	82	75	66	71	Too soft texture inside if golden crispy crust—replace with another ingredient	Change pumpkin to cauliflower
**Thai Beef Salad (*n* = 10)**	72	26	34	59	Overwhelming umami, few participants would make this at home	Discard
**Meatballs in Curry (*n* = 10)**	84	82	86	93	Well-liked, can be optimised with taste and texture	Modify and reuse in different versions at next class
**Fried Rice with Eggs (*n* = 10)**	70	48	55	61	Okay, but parosmic trigger in three participants (all rarely experience parosmia)	Discard

**Table 3:** Improvements of the CAFPAS questions after the cooking school.

	Normosmic Reference (*n* = 166)	Before Cooking School (*n* = 48)	End of Cooking School (*n* = 48)	Δ	*p*-Value
**Prefer to cook rather than having food prepared**	4.31	3.49	4.23	0.74	0.0074
**Cooking is a fulfilling activity**	5.70	5.02	5.48	0.46	0.0245
**Confidence in choosing between similar products**	5.93	5.50	5.96	0.46	0.0166
**Confidence in ability to deal with unexpected results during cooking**	5.38	4.73	5.13	0.40	0.1896
**Involvement in daily meal preparations**	6.15	5.94	6.27	0.33	0.0250

Normative scores from Fjaeldstad and Smith, 2022 [13]. For detailed information on the questions, see the original publication by Lahne et al. [21]. For a complete list of changes in CAFPAS questions, see Table A3.

## Data Availability

The original contributions presented in the study are included in the article, further inquiries can be directed to the corresponding author.

## Appendix A

Supplemental information on the design and contents of the cooking school. This additional information is included to enable other clinics to design similar rehabilitation efforts for patients with smell loss. For additional questions, contact the corresponding author.

**Table A1:** Setup of the five-week cooking school.

Session	Contents
**Session 1** **(−1 day)**	- Olfactory and gustatory testing (TDI and taste spray/taste drop test) - Questionnaires on taste/smell, QoL, and food/cooking habits
**Session 1**	- Introduction to the cooking school, smell loss, and current knowledge on cooking with smell loss (researcher and ENT physician) - Introduction to the concept of the cooking school, including 5T’s, taste kit, and texture bank (chef) - ‘Sensory Circus’ with tasting samples and description of all senses involved in the perception of food (chef) - Meal prepared by chefs and social dining - Availability of ENT physician if participants have questions regarding smell loss and treatment - Box with printed cookbook [24], taste kit, and texture bank to take home
**Session 2–3**	- Short description of the 5T’s, seasoning with the taste kit, texture, and guidance on how to use the kitchen - Cooking (recipes) in small teams based on TDI scores - Guidance by chefs during cooking and seasoning - Presentation of food samples using three levels of seasoning (1: salt and pepper; 2: added balanced seasoning of all five basic tastes; 3: added different textures and visual presentation) [24] - Presentation of dishes and usage of the 5T’s in plenum - Social dining and evaluation of dishes and recipes
**Session 4**	- Presentation of nutritional aspects and challenges in smell loss (clinical dietitian) - Identical contents as session 2–3
**Session 5**	- Participants were encouraged to bring a relative to join the cooking school on the final day. - Short description of the 5T’s, seasoning with the taste kit, texture, and guidance on how to use the kitchen - “Food Jam”, where participants created a dish using limited available ingredients and learned methods without being handed a recipe. Continuous feedback from chef during the preparation process - Presentation of created dishes to the class before social dining, with emphasis on applied methods and used ingredients. - Social dining and evaluation
**Session 5** **(+0 days)**	- Online individual evaluation of the cooking school - Questionnaires on taste/smell, QoL, and food/cooking habits
**Session 5** **(+90 days)**	- Questionnaires on taste/smell, QoL, and food/cooking habits

**Table A2:** Pedagogical goals for participants and applied methods during the cooking school [57].

Pedagogical Goals	Applied Methods
**Acquire an understanding of own sensory abilities**	- Pre-test of measured taste (taste spray/taste drop test) and smell scores (Sniffin’ Sticks TDI score) - Sensory challenge (day one) using food samples to illustrate how other senses still give sensory stimulation in food.
2. **Acquire a basic knowledge on all senses involved in the multisensory experience of eating**	- Sensory challenge (day 1), where oral and visual information was combined with food samples to illustrate the perception of basic tastes, smell, texture, colours, mouthfeel, spiciness, and multisensory integration. - Introductory lecture on all senses relevant for flavour perception (day 1) - A printed version of the cooking school cookbook was given to all participants (day one) [24] - Presentation of food samples using three levels of seasoning (1: salt and pepper; 2: added balanced seasoning of all five basic tastes; 3: added different textures and visual presentation) [24]
3. **Improve cooking and seasoning abilities**	- Practicing seasoning with “The 5T’s” (see Figure 1) - Practicing achieving a balance of basic tastants [58,59] - Introduction to the sensory qualities of new foods and seasoning products to increase knowledge and repertoire of food items in their cooking.
4. **Build confidence in cooking abilities**	- Cooking preparation in teams (*n* = 2–3) with continuous feedback from chef during the preparation process - Presentation of created dishes to the class before social dining (day 2–5), with emphasis on applied methods and used ingredients. - “Food Jam” (day 5), where participants created a dish using limited available ingredients and learned methods without being handed a recipe.
5. **Transfer of cooking school contents to own kitchen**	- Encouragement to use ‘taste kit’ and methods at home between sessions. - Participants were encouraged to bring a relative to join the cooking school on the final day.
6. **Social belonging**	- Social dining (day 1–5), where all participants and staff shared the produced dishes at the same table. - Meeting others with similar olfactory deficits and discussing experiences and coping mechanisms during cooking - Presence of physician and researcher experienced in working with smell loss to demystify any general questions on smell loss in a relaxed setting (day 1 and 5)

## Appendix B

**Table A3:** Differences in cooking and food provisioning habits between normosmics and cooking school patients.

	Normosmic Reference (*n* = 166)	Start of Cooking School (*n* = 48)	End of Cooking School (*n* = 47)	Δ	*p*-Value
**Want to get through cooking ASAP. (FSE14 (neg))**	2.58 (2.28; 2.88)	3.33 (2.82; 3.85)	3.17 (2.67; 3.67)	−0.16	0.3305
**Cooking is a fulfilling activity (FSE11)**	5.70 (5.42; 5.98)	5.02 (4.48; 5.56)	5.48 (5.06; 5.89)	0.46	0.0245
**Comfortable preparing food (ISCO1)**	6.21 (5.95; 6.47)	5.58 (5.12; 6.05)	5.69 (5.34; 6.04)	0.11	0.5924
**Inspired to cook for other people (STR2)**	5.67 (5.39; 5.96)	5.34 (4.84; 5.84)	5.53 (5.11; 5.96)	0.19	0.1725
**Easy to accomplish desired results during cooking (FSE7)**	5.67 (5.42; 5.93)	4.75 (4.29; 5.21)	4.85 (4.48; 5.22)	0.10	0.4784
**Cooking is a waste of effort (ISCU4 (neg))**	1.89 (1.64; 2.15)	2.15 (2.54; 3.05)	1.77 (1.46; 2.01)	−0.38	0.0626
**Will not make a new dish again after unsuccessful first attempt (FSE16 (neg))**	3.43 (3.16; 3.71)	4.08 (3.58; 4.59)	3.83 (3.25; 4.41)	−0.25	0.2784
**Cooking brings little enjoyment (FSE15 (neg))**	3.31 (3.02; 3.61)	4.60 (4.14; 5.07)	4.53 (4.10; 4.96)	−0.07	0.7644
**Confidence in ability to deal with unexpected results during cooking (FSE6)**	5.38 (5.11; 5.64)	4.73 (4.18; 5.28)	5.13 (4.73; 5.52)	0.40	0.1896
**Ability to decide what to eat (FSE3)**	4.99 (4.70; 5.28)	4.85 (4.35; 5.36)	4.63 (4.16; 5.09)	−0.22	0.5771
**Cooking for others is a burden (STR3 (neg))**	2.57 (2.28; 2.85)	2.71 (2.17; 3.24)	2.52 (2.08; 2.96)	−0.19	0.2713
**Coping with problems during cooking (FSE8)**	5.67 (5.43; 5.92)	5.06 (4.58; 5.55)	5.15 (4.76; 5.54)	0.09	0.6248
**Prefer to spend time on more important things than food (HO5 (neg))**	3.20 (2.92; 3.47)	3.60 (3.11; 4.09)	3.15 (2.71; 3.58)	−0.55	0.0371
**Limited by lack of cooking knowledge (FSE2 (neg))**	2.36 (2.07; 2.65)	2.73 (2.22; 3.24)	3.13 (2.62; 3.63)	0.40	0.1714
**Involvement in daily meal preparations (ISMP2)**	6.15 (5.88; 6.42)	5.94 (5.45; 6.42)	6.27 (5.90; 6.64)	0.33	0.0250
**Prefer to cook rather than having food prepared (ISCO3)**	4.31 (4.01; 4.61)	3.49 (2.96; 4.02)	4.23 (3.76; 4.70)	0.74	0.0074
**Confidence in creating meals from ingredients on hand (ISMP5)**	6.04 (5.81; 6.28)	5.64 (5.18; 6.10)	6.02 (5.75; 6.29)	0.38	0.0867
**Confidence in choosing between similar products (ISSH4)**	5.93 (5.71; 6.16)	5.50 (5.09; 5.91)	5.96 (5.73; 6.18)	0.46	0.0166
**Reflection on what to cook and eat (HO4)**	5.14 (4.88; 5.41)	4.60 (4.19; 5.00)	4.96 (4.60; 5.32)	0.36	0.0711
**Knowledge of usage of ingredients during purchasing (ISMP3)**	6.17 (5.97; 6.38)	5.89 (5.50; 6.29)	5.98 (5.71; 6.24)	0.11	0.5062
**Difficult finding time to prepare preferred food (STR4 (neg))**	3.63 (3.35; 3.90)	2.94 (2.39; 3.48)	3.09 (2.56; 3.61)	0.15	0.2196
**Knowledge on where to find needed ingredients (ISSH5)**	6.34 (6.15; 6.53)	6.10 (5.75; 6.46)	6.23 (5.95; 6.51)	0.13	0.2424
**No time to prepare meals due to family responsibilities (STR8 (neg))**	2.62 (2.36; 2.88)	2.40 (1.92; 2.87)	2.33 (1.86; 2.75)	−0.07	0.8165
**No time to prepare meals due to job responsibilities (STR10 (neg))**	3.31 (3.00; 3.62)	2.35 (1.82; 2.89)	2.23 (1.78; 2.68)	−0.12	0.5418
**Knowledge of kitchen equipment usage (ISCO5)**	6.37 (6.19; 6.56)	6.15 (5.76; 6.54)	6.31 (6.06; 6.56)	0.16	0.3375
**Mental plan of steps before cooking (ISMP7)**	5.82 (5.59; 6.05)	5.71 (5.31; 6.11)	5.92 (5.58; 6.25)	0.21	0.3021
**No time to prepare meals due to social responsibilities (STR9 (neg))**	2.43 (2.19; 2.66)	2.17 (1.71; 2.63)	2.11 (1.73; 2.48)	−0.06	0.8167
**Wish for more time to plan meals (ISMP1 (neg))**	3.98 (3.73; 4.24)	3.48 (2.94; 4.01)	3.48 (2.94; 4.01	0.00	0.9070
